# Optimal surgical management of unifocal vs. multifocal NF-PNETs: a respective cohort study

**DOI:** 10.1186/s12957-024-03383-9

**Published:** 2024-04-26

**Authors:** Juwan Kim, Seung Soo Hong, Sung Hyun Kim, Ho Kyong Hwang, Chang Moo Kang

**Affiliations:** 1 Department of surgery, Yonsei University College of Medicine, Seoul, Korea; 2 Division of Hepatobiliary and Pancreatic Surgery, Department of surgery, Yonsei University College of Medicine, Seoul, Korea; 3 Pancreatobiliary Cancer Center, Yonsei Cancer Center, Severance Hospital, Seoul, Korea

**Keywords:** Gastro-enteropancreatic neuroendocrine tumor, Multifocality, Pancreatectomy, Recurrence, Postoperative complications

## Abstract

**Background:**

Pancreatic neuroendocrine tumors (PNETs) represent 1–2% of pancreatic tumors, with recent guidelines recommending active surveillance for non-functioning PNETs (NF-PNETs) smaller than 2 cm. However, the management of multiple NF-PNETs, as well as the influence of tumor number on prognosis, remains under-researched.

**Methods:**

This retrospective study analyzed NF-PNET patients who underwent pancreatic resection at Severance Hospital between February 1993 and August 2023, comparing the characteristics of patients diagnosed with multifocal tumors and those with unifocal tumors. A subgroup analysis of overall survival (OS) and recurrence-free survival (RFS) was performed based on multifocality employing the Kaplan-Meier method and the log-rank test.

**Results:**

Of 187 patients, 169 (90.4%) had unifocal and 18 (9.6%) had multifocal tumors. Multifocal tumors were more likely to be diffusely spread, necessitating more total pancreatectomies (diffuse tumor location: 4.7% in unifocal vs. 38.9% in multifocal cases, *p* < 0.001; total pancreatectomy: 4.1% in unifocal vs. 33.3% in multifocal cases, *p* < 0.001). In patients with NF-PNET who underwent the same extent of pancreatic resection, no significant difference in the incidence of complication was observed regardless of multifocality. Moreover, no significant difference in OS was seen between the unifocal and multifocal groups (log-rank test: *p* = 0.93). However, the multifocal group exhibited a poorer prognosis in terms of RFS compared to the unifocal group (log-rank test: *p* = 0.004) Hereditary syndrome, tumor grade, size, lymphovascular invasion, and lymph node metastasis were key factors in the recurrence.

**Conclusion:**

This study’s findings suggest that the presence of multiple tumors was associated with poorer recurrence-free survival but did not affect long-term survival following surgery. Given the long-term oncologic outcome and quality of life following surgery, resection of tumors over 2 cm is advisable in patients with multifocal PNETs, while a cautious “wait-and-see” approach for smaller tumors (under 2 cm) can minimize the extent of resection and improve the quality of life. In cases with only small multifocal NF-PNETs (< 2 cm), immediate resection may not be crucial, but the higher recurrence rate than that in solitary NF-PNET necessitates intensified surveillance.

**Supplementary Information:**

The online version contains supplementary material available at 10.1186/s12957-024-03383-9.

## Background

Pancreatic neuroendocrine tumors (PNETs) are rare, comprising only 1–2% of all pancreatic tumors [1]. Despite their rarity, instances of early-stage PNET diagnoses have risen considerably in recent years [2], primarily due to the incidental discovery of non-functioning PNETs (NF-PNETs) [2]. Distinguishing between functional and non-functional PNETs is crucial, since their surgical interventions can vary significantly [3]. For instance, unlike their functional counterparts, NF-PNETs do not necessitate surgery for a biochemical cure, complicating the decision-making process for surgical intervention [3]. 

For sporadic NF-PNETs measuring less than 2 cm, both current North American Neuroendocrine Tumor Society Consensus and European Neuroendocrine Tumor Society (ENETS) guidelines recommend active surveillance [3–5]. Previous studies support this approach, showing a stable prognosis without surgery [6–9]. Nevertheless, surgical treatment is the primary intervention for NF-PNETs, as it is crucial for survival as well as in preventing disease recurrence. While there has been some consensus on surgical indications and methods for solitary tumors, there is still insufficient research on these aspects when dealing with multiple tumors.

Multiple tumors are often associated with syndromic PNETs, such as those seen in Multiple Endocrine Neoplasia type 1 (MEN1) patients or Von Hippel-Lindau syndrome (VHL syndrome), which tend to occur at a younger age and frequently manifest as multifocal growths [10, 11]. When tumors are multifocal, achieving complete resection becomes challenging, and an extensive surgical approach might increase the risk of complications and negatively impact the patient’s quality of life [12, 13]. Therefore, it is essential to carefully consider various factors, including oncological outcomes, complications, and postoperative quality of life when determining the appropriate management [3, 4]. In previous research comparing the survival rates of multifocal tumors to unifocal tumors in PNET, only functioning PNETs were included [14]. Therefore, no study so far has directly investigated long-term survival and complications based on the number of NF-PNETs.

Hence, this study aimed to investigate possible differences in overall survival and recurrence between unifocal and multifocal tumors in patients diagnosed with NF-PNETs. In addition, we aimed to determine the differences in surgical approaches and complications between patients NF-PNETs who underwent surgery for solitary tumors and those with multiple tumors.

## Method

### Study population and data collection

A retrospective study was conducted on patients who were pathologically diagnosed with NF-PNET at Severance Hospital from February 1993 to August 2023. Those with NF-PNETs showed no biochemical evidence and underwent pancreatic resection at our institution. This study was approved by the Institutional Review Board (IRB) of our institution (Approval Number: 2023-2536-001). Patient records were thoroughly reviewed, including imaging findings, radiology reports, surgical records, pathology reports, post-operative follow-up, and outpatient records. This review aimed to verify patients’ baseline characteristics, tumor size and features, type of surgery, surgical complications, and long-term outcomes like recurrence and survival.

### Diagnostic criteria for hereditary syndromes

Patients diagnosed with MEN1 were identified based on the international diagnostic guideline for this disease [15]. These patients either met the clinical diagnostic criteria, which necessitates diagnosis with two or more MEN1-related endocrine tumors; satisfied the familial diagnostic criteria wherein a first-degree relative was diagnosed with MEN1 and presented with a MEN1-associated tumor; or met the genetic diagnosis criteria implying the discovery of a mutation in the MEN1 gene upon genetic testing. Patients diagnosed with VHL either exhibited two or more VHL-related clinical symptoms including hemangioblastoma, or presented a family history of VHL along with a mutation in the VHL gene and at least one VHL-related clinical symptom [16]. 

### Tumor classification and assessment criteria

The tumor grade was classified using the World Health Organization (WHO) grade system from 2022 [17, 18]. The staging of the NET was determined based on the 8th edition of the American Joint Committee on Cancer staging system [19]. Multifocality was defined based on the number of tumors found in pathological results after surgery; cases with two or more tumors were considered multifocal [20]. Pancreatic neuroendocrine microadenomas (NEMA), as observed in pathological findings, were also included in the determination of multifocality [21]. Tumor location was classified as follows: “head” referred to the right side of the left border of the Superior Mesenteric Vein (SMV); “body” was the region between the left border of the SMV and the left border of the aorta; and “tail” denoted the section from the aorta’s left border to the spleen hilum. Tumors spanning more than two of these regions were categorized as “diffuse.” The size of the tumor was determined by its largest diameter as identified through pathological findings. Both lymph node metastasis and distant metastasis were determined based on pathological criteria.

### Perioperative and postoperative data analysis

Our study involved a comprehensive collection of perioperative and postoperative data by reviewing various medical records, including surgical, anesthesia, and postoperative progress notes. We assessed patients’ preoperative condition by collecting data such as Body Mass Index (BMI) and American Society of Anesthesiologists (ASA) score from their medical records [22]. The types of surgery and approaching method (open surgery vs. minimally invasive surgery (MIS)) were collected by referring to the surgical records. Open surgery included both direct open surgical procedures and those initially attempted as minimally invasive surgery but converted to open surgery. The MIS encompassed both laparoscopic and robotic surgical procedures. Intraoperative data included the duration of surgery, the volume of blood loss, and the necessity for blood transfusions. These details were obtained from the surgical and anesthesia records. Postoperative outcomes were analyzed through postoperative progress notes and relevant test results, encompassing the duration of hospital stay post-surgery and the incidence of any complications. Complications were sorted according to the Clavien–Dindo classification, emphasizing the highest grade of complication experienced by each patient [23]. Additionally, the Comprehensive Complication Index (CCI) was utilized to identify and assess the presence of concurrent complications [24]. The occurrences of Delayed Gastric Emptying (DGE) and Postoperative Pancreatic Fistula (POPF) were evaluated in accordance with the International Study Group of Pancreatic Surgery (ISGPS) classification system [25–28]. The tumor burden score was calculated as the square root of the sum of the squares of both the size and the number of tumors, following the methodology proposed in previous studies [29]. 

### Statistical analysis

We conducted a comparative analysis between patients diagnosed with multifocal tumors and those with unifocal tumors. Categorical variables were compared using the χ2 Test and Fisher’s exact test. For continuous variables, after performing a normality test, we used the t-test or the Mann–Whitney U test. In our institution, patients with sporadic PNETs were followed up every 3–6 months for the first 1–2 years after surgery, then annually for up to 5 years. Patients with hereditary syndromes were also followed up every 3–6 months immediately after surgery, and then underwent lifelong imaging surveillance once every 1–2 years after the first year. Overall survival (OS) was determined from the surgery date up to the event occurrence date. Recurrence-free survival (RFS) was defined based from the date of the surgery up to the date when a recurrence was confirmed during an outpatient follow-up post-surgery. The Cox model was used to evaluate the hazard ratio (HR) of the risk factors in respect of OS and RFS. Variables that demonstrated statistical significance, with a P-value < 0.05 for RFS and a P-value < 0.2 for OS, were included in multiple regression analyses, respectively. We then performed stepwise regression using the forward conditional method. The Kaplan Meier method and log-rank test was used to evaluate the difference between patients with multifocal tumor and unifocal tumor. In addition, we conducted a survival analysis on RFS in patients with small NF-PNETs (less than 2 cm) excluding those with VHL syndrome, comparing unifocal tumor to multifocal tumors.

A P-value < 0.05 was deemed statistically significant. All statistical analyses were executed using SPSS Version 24 (IBM, Chicago, IL) and R.3.6.3.

## Result

### Patient demographics

Between February 1993 and August 2023, a total of 187 patients at Severance Hospital were pathologically diagnosed with NF-PNETs and underwent surgery. Among these, 169 patients (90.4%) were diagnosed with a single tumor, while 18 patients (9.6%) presented with multiple tumors. The baseline characteristics of patients with single and multiple tumors are presented in Table 1.

**Table 1:** Baseline characteristics of patients with unifocal and Multifocal NF-PNETs

Variables	Unifocal	Multifocal	Total	p
	(*N* = 169)	(*N* = 18)	(*N* = 187)	
Number of tumors, median	1.0 [1.0;1.0]	4.0[3.0;4.0]	1.0 [1.0;1.0]	< 0.001
Bifocal	-	4 (22.2%)	-	
More than 2	-	14 (77.8%)	-	
Male: Female	82:87 (48.5%:51.5%)	7:11 (38.9%:61.1%)	89:98 (47.6%:52.4%)	0.596
BMI (kg/m²), mean	24.2 ± 3.3	22.3 ± 3.4	24.0 ± 3.3	0.027
ASA score				0.187
I	34 (20.1%)	7 (38.9%)	41 (21.9%)	
II	100 (59.2%)	8 (44.4%)	108 (57.8%)	
III	35 (20.7%)	3 (16.7%)	38 (20.3%)	
Sporadic vs. hereditary PNET				< 0.001
Sporadic	163 (96.4%)	9 (50.0%)	172 (92.0%)	
MEN1	4 (2.4%)	6 (33.3%)	10 (5.3%)	
VHL	2 (1.2%)	3 (16.7%)	5 (2.7%)	
Tumor location				< 0.001
Head	61 (36.1%)	2 (11.1%)	63 (33.7%)	
Body	47 (27.8%)	2 (11.1%)	49 (26.2%)	
Tail	53 (31.4%)	7 (38.9%)	60 (32.1%)	
Diffuse	8 (4.7%)	7 (38.9%)	15 (8.0%)	
Minimally invasive approach	105 (62.1%)	11 (61.1%)	116 (62.0%)	> 0.999
Type of surgery				< 0.001
Enucleation	24 (14.2%)	0 (0.0%)	24 (12.8%)	
Distal pancreatectomy	84 (49.7%)	9 (50.0%)	93 (49.7%)	
Central pancreatectomy	8 (4.7%)	1 (5.6%)	9 (4.8%)	
PPPD	46 (27.2%)	2 (11.1%)	48 (25.7%)	
Total pancreatectomy	7 (4.1%)	6 (33.3%)	13 (7.0%)	
WHO grade				0.481
Grade 1	128 (75.7%)	12 (66.7%)	140 (74.9%)	
Grade 2	38 (22.5%)	5 (27.8%)	43 (23.0%)	
Grade 3	3 (1.8%)	1 (5.6%)	4 (2.1%)	
Largest tumor size (cm), mean	2.5 ± 2.0	2.8 ± 2.2	2.6 ± 2.0	0.547
Lymph node metastasis	16 (9.5%)	5 (27.8%)	21 (11.2%)	0.052
Distant metastasis	2 (1.2%)	1 (5.6%)	3 (1.6%)	0.677
AJCC 8th stage				0.212
Stage 1	73 (43.2%)	6 (33.3%)	79 (42.2%)	
Stage 2	78 (46.2%)	7 (38.9%)	85 (45.5%)	
Stage 3	15 (8.9%)	4 (22.2%)	19 (10.2%)	
Stage 4	3 (1.8%)	1 (5.6%)	4 (2.1%)	

BMI, body mass index; ASA, American Society of Anesthesiologists; MEN1, multiple endocrine neoplasm type 1; PNET, pancreatic neuroendocrine tumor; VHL, Von Hippel-Lindau syndrome; PPPD, pylorus-preserving pancreaticoduodenectomy; WHO, World Health Organization; AJCC, American Joint Committee on Cancer

Of those with multiple tumors, four patients (4/18, 22.2%) had two tumors, and 14 patients (14/18, 77.8%) had three or more tumors. Fifteen patients (8.0%) were diagnosed with hereditary syndrome and 10 patients (10/187, 5.3%) had MEN1, accounting for 5.3% of the total cohort, and a further five patients (5/187, 2.7%) were diagnosed with VHL disease.

The majority of unifocal PNET patients (96.4%) were diagnosed with sporadic PNET. In contrast, 50% of multifocal PNET patients (nine out of 18) were diagnosed with a hereditary syndrome (*p* < 0.001). When comparing the tumor characteristics between unifocal and multifocal groups, the latter tended to be more diffusely spread, and the rate of total pancreatectomy was higher in this group (tumor location: diffuse, 4.7% in unifocal PNET patients vs. 38.9% in the patients whose tumor was multifocal, *p* < 0.001. Of the patients requiring total pancreatectomy, 4.1% were in the unifocal group vs. 33.3% in the multifocal group, *p* < 0.001).

No significant differences were observed between the two groups in terms of WHO grade or size of the largest tumor. However, lymph node metastasis was more prevalent in patients with multifocal tumors (27.8%) compared to those with unifocal tumors (9.5%), suggesting a trend towards higher incidence (*p* = 0.052).

In the study population, we examined the differences in postoperative outcomes and complications between MIS and open surgery. As shown in (Supplementary Table 2), there were significant differences in the estimated blood loss and the length of hospital stay between the two groups, with no differences observed in other measures. Specifically, the estimated blood loss was 250.0 [100.0; 600.0] for MIS versus 100.0 [50.0; 200.0] for open surgery (*p* < 0.001), and the length of hospital stay was 14.0 [11.0; 25.5] days for MIS compared to 9.0 [8.0; 13.0] days for open surgery (*p* < 0.001).

### Comparative analysis of surgical outcomes in multifocal vs. unifocal tumor patients

We investigated the differences between patients diagnosed with multifocal tumors and those with unifocal tumors by categorizing them based on the types of surgery they underwent shown as Table 2. Patients who underwent enucleation and distal pancreatectomy were classified under minor resection. The baseline characteristics revealed that patients with multifocal tumors tended to be younger, and the prevalence of hereditary syndrome was higher compared to those with a unifocal tumor (53.7% vs. 39.6%, *p* = 0.002; 2.8% vs. 33.3%, *p* = 0.001). No significant differences were observed between the two groups in the other baseline characteristics. Regarding surgical details such as operation time, blood loss, postoperative complications, and hospital stay duration, no significant differences were noted between the two groups. Patients who received either PPPD (Pylorus-Preserving Pancreatico-duodenectomy) or central pancreatectomy, which were categorized as a major resection, also showed no significant differences in their baseline characteristics, surgical details, postoperative complications, and duration of hospital stay when compared with the groups with multifocal and unifocal tumors. Similarly, for patients who underwent total pancreatectomy, no significant differences were observed in surgical details, postoperative complications, and hospital stay duration between the two groups.

**Table 2:** Comparison of perioperative outcomes between patients with unifocal NF-PNETs and multifocal NF-PNETs according to type of surgery

	Minor resection (Enucleation, DP)			Major resection (PPPD, CP)			Total pancreatectomy		
	**Unifocal**	**Multifocal**	p	**Unifocal**	**Multifocal**	p	**Unifocal**	**Multifocal**	p
	(*N* = 108)	(*N* = 9)		(*N* = 54)	(*N* = 3)		(*N* = 7)	(*N* = 6)	
Type of surgery	Enuc: 24 (22.2%)	DP: 9 (100.0%)	0.247	PPPD: 46 (85.2%)	PPPD: 2 (66.7%)	0.966	-		
	DP: 84 (77.8%)			CP: 18 (14.8%)	CP:1 (33.3%)				
MIS	75 (69.4%)	7 (77.8%)	0.884	27 (50.0%)	0 (0.0%)	0.274	3 (42.9%)	4 (66.7%)	0.764
Male	57 (52.8%)	3 (33.3%)	0.439	21 (38.9%)	2 (66.7%)	0.726	4 (57.1%)	2 (33.3%)	0.764
Age, mean	53.7 ± 12.3	39.6 ± 15.7	0.002	57.0 [43.0;65.0]	47.0 [45.5;48.5]	0.231	51.1 ± 11.8	43.5 ± 6.1	0.181
BMI (kg/m²), mean	24.4 ± 3.2	22.7 ± 3.8	0.135	23.0 [21.8;25.9]	24.3 [23.9;25.4]	0.421	22.8 ± 3.4	20.6 ± 3.0	0.243
ASA score			0.142			0.319			0.692
	27 (25.0%)	5 (55.6%)		5 (9.3%)	1 (33.3%)		2 (28.6%)	1 (16.7%)	
	60 (55.6%)	3 (33.3%)		36 (66.7%)	2 (66.7%)		4 (57.1%)	3 (50.0%)	
	21 (19.4%)	1 (11.1%)		13 (24.1%)	0 (0.0%)		1 (14.3%)	2 (33.3%)	
Hereditary SD	3 (2.8%)	3 (33.3%)	0.001	2 (3.7%)	0 (0.0%)	> 0.999	1 (14.3%)	6 (100.0%)	0.011
OP time (min), median	184.5 [141.0;244.5]	185.0 [169.0;234.0]	0.79	398.5 ± 112.7	384.7 ± 136.4	0.838	407.0 [382.0;504.5]	432.5 [375.0;526.0]	> 0.999
Blood Loss(cc), median	100.0 [30.0;200.0]	50.0 [20.0;100.0]	0.361	275.0 [100.0;550.0]	550.0 [290.0;775.0]	0.844	400.0 [325.0;950.0]	325.0 [200.0;350.0]	0.222
Transfusion	4 (3.7%)	0 (0.0%)	> 0.999	6 (11.1%)	0 (0.0%)	> 0.999	3 (42.9%)	0 (0.0%)	0.243
Postop complication	41 (38.9%)	2 (22.2%)	0.526	36 (66.7%)	2 (66.7%)	> 0.999	3 (42.9%)	4 (66.7%)	0.764
DGE			0.952			0.966			0.188
Grade A	5 (4.6%)	1 (11.1%)		8 (14.8%)	1 (33.3%)		0 (0.0%)	2 (33.3%)	
Grade B	-	-		-	-		1 (14.3%)	0 (0.0%)	
POPF			0.316			0.852	-	-	
Grade A	36 (33.3%)	1 (11.1%)		18 (33.3%)	1 (33.3%)		-	-	
Grade B	5 (4.6%)	1 (11.1%)		5 (9.3%)	0 (0.0%)		-	-	
CCI, median	0.0 [0.0; 8.7]	0.0 [0.0; 0.0]	0.402	8.7 [0.0;20.9]	8.7 [4.3;14.8]	0.726	0.0 [0.0; 8.7]	8.7 [0.0;20.9]	0.492
clavien dindo (Highest grade)			0.521			0.958			0.307
1	20 (18.5%)	1 (11.1%)		12 (22.2%)	1 (33.3%)		2 (28.6%)	2 (33.3%)	
2	15 (13.9%)	0 (0.0%)		11 (20.4%)	1 (33.3%)		-	2 (33.3%)	
3a	6 (5.6%)	1 (11.1%)		7 (13.0%)	0 (0.0%)		-	-	
3b	-	-		3 (5.6%)	0 (0.0%)		-	-	
4a	-	-		2 (3.7%)	0 (0.0%)		1 (14.3%)	-	
4b	-	-		-	-		-	-	
PostOp LOS (days), median	9.0 [8.0;13.0]	10.0 [7.0;14.0]	0.746	14.5 [11.0;27.0]	20.0 [15.5;25.5]	0.641	20.0 [11.0;28.5]	14.0 [13.0;15.0]	0.943

DP, Distal pancreatectomy; PPPD, Pylorus-preserving pancreaticoduodenectomy; CP, Central pancreatectomy; Enuc, Enucleation; MIS, Minimally invasive surgery; BMI, Body mass index; ASA, American Society of Anesthesiologists; Hereditary SD, Hereditary syndrome; OP time, operation time; postop complication, postoperative complication; DGE, Delayed Gastric Emptying; POPF, Postoperative pancreatic fistula; CCI, Comprehensive Complication Index; Clavien Dindo, Cavien Dindo classification; PostOp LOS, Postoperative length of stay

### Long-term oncologic impact of multifocality and determining other risk factors in patients with NF-PNETs

The median follow-up duration for these patients was 52.7 months [22.1; 85.5].

**Fig. 1:**
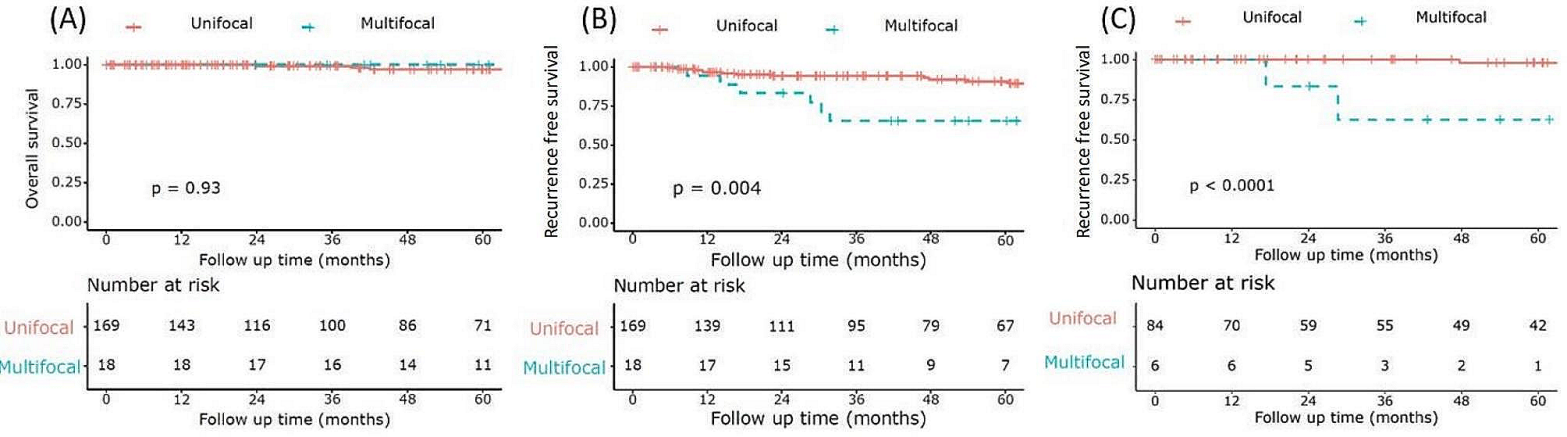
Kaplan-Meier curves of NF-PNET patient comparisons between unifocal and multifocal tumors. (**A**) Overall survival and (**B**) recurrence-free survival in overall patients, (**C**) recurrence-free survival in subgroup analysis for NF-PNETs < 2 cm except patients with Von Hippel-Lindau syndrome

There was no significant difference in OS between the unifocal and multifocal groups (log-rank test: *p* = 0.93, Fig. 1-A). However, the multifocal group exhibited a poorer prognosis in terms of RFS compared to the unifocal group (log-rank test: *p* = 0.004, Fig. 1-B). The patterns of recurrence following resection in each group are summarized in (Supplementary Table 2).

Statistically significant differences in OS were found in relation to the WHO grade, with grade 3 having a HR of 25.704 for OS (95% CI: 2.426–272.393, *p* = 0.007), using grade 1 as a reference. The presence of a hereditary syndrome and the number of tumors did not show a significant impact on OS. Other variables also did not demonstrate significant differences in OS.

Regarding recurrence after surgery, hereditary SD (HR: 3.132, 95% CI: 1.133–8.657, *p* = 0.028); WHO grade 2 (HR: 4.032, 95% CI: 1.589–10.233, *p* = 0.003); WHO grade 3 (HR: 22.405, 95% CI: 4.661–107.692, *p* < 0.001); multifocality (HR: 3.716, 95% CI: 1.425–9.686, *p* = 0.007); tumor size over 2 cm (HR: 4.357, 95% CI: 1.452–13.077, *p* = 0.009); lymphovascular invasion (HR: 4.135, 95% CI: 1.582–10.811, *p* = 0.004); and lymph node metastasis (HR: 3.424, 95% CI: 1.239–9.458, *p* = 0.018) were all significantly associated with recurrence. In addition, the tumor burden score also showed a significant association with recurrence (HR:1.232, 95% CI: 1.072–1.415, *p* = 0.003.) Other factors did not show a significant difference (Table 3). The results of multivariable analysis in OS, RFS are shown in (supplementary Figs. 1, 2).

**Table 3:** Determining risk factors of overall survival and recurrent-free survival in patients with NF-PNETs

	Overall survival		Recurrence-free survival	
Variables	HR (95% CI)	p	HR (95% CI)	P
Male	1.05 (0.258–4.268)	0.946	1.299 (0.538–3.138)	0.561
(ref: female)				
Age over 65	3.235 (0.720–14.543)	0.126	0.812 (0.237–2.785)	0.741
(ref: ≤65)				
Hereditary PNETs	0.727(0.087–6.099)	0.77	3.132 (1.133–8.657)	0.028
(ref: sporadic)				
Minimally invasive approach	1.373 (0.294–6.414)	0.687	0.503 (0.2–1.266)	0.145
(ref: open surgery)				
Positive resection margin	1.305 (0.157–10.866)	0.806	1.783 (0.522–6.091)	0.356
(ref: R0)				
WHO grade2	2.519 (0.507–12.504)	0.2584	4.032 (1.589–10.233)	0.003
(ref: G1)				
WHO grade3	25.704 (2.426–272.393)	0.007	22.405 (4.661–107.692)	> 0.001
(ref: G1)				
Multifocal	1.092 (0.131–9.108)	0.935	3.716 (1.425–9.686)	0.007
(ref: unifocal)				
Tumor size > 2 cm	3.695 (0.741–18.431)	0.111	4.357 (1.452–13.077)	0.009
(ref: ≤2 cm)				
Tumor Burden Score (continuous)	1.085 (0.828–1.422)	0.556	1.232 (1.415–1.072)	0.003
Lymphovascular invasion	3.929 (0.757–20.396)	0.104	4.135 (1.582–10.811)	0.004
Lymph node metastasis	unbounded	–	3.424 (1.239–9.458)	0.018

Cox proportional hazard model was used to determine the risk factors for overall survival and recurrent-free survival. HR, PNETs, pancreatic neuroendocrine tumors; WHO, World Health Organization; G1, grade 1; 95% CI, confidential interval

When investigating the difference between patients with unifocal tumors and multifocal tumors in subgroups who were diagnosed with small NF-PNET (less than 2 cm) except for the VHL syndrome, the multifocal group showed poorer RFS compared to the unifocal group (log-rank test: *p* < 0.001, Fig. 1-C). However, there was no significant difference in OS (log-rank test: *p* = 0.82).

## Discussion

The primary aim of this study was to determine whether patients with NF-PNETs who have multifocal tumors exhibit worse OS or RFS compared to those with unifocal tumors. After analyzing its impact on long-term prognosis, our study found that multifocality did not show a difference in OS, however, when compared with unifocal tumors there was a difference in RFS.

A review of relevant studies yields several noteworthy insights. An initial retrospective study from a single institution focusing on patients with sporadic PNETs, including those with functional PNETs, found no differences in OS and RFS when comparing patients based on the number of tumors [14]. In contrast, a separate multicenter study highlighted a significant link between the Tumor Burden Score and the recurrence rate in patients with NF-PNETs [29]. The Tumor Burden Score, calculated as the square root of the sum of the squares of the tumor size and the number of tumors, implies that the number of tumors might impact prognostic findings. In this study, we also confirmed that the tumor burden score itself can be a prognostic factor for predicting the recurrence rate of NF-PNETs. These divergent findings underscore the need for more in-depth research into how the number of tumors affects long-term outcomes. Conducting such studies is crucial for developing accurate and effective clinical strategies for managing patients diagnosed with multifocal NF-PNETs.

The secondary aim of our study was to compare surgical outcomes between patients with multifocal PNETs and those with unifocal PNET, and particularly to determine possible differences in surgery-related outcomes when both groups undergo the same procedures for pancreatic resection. The results indicated that, despite the difference in multifocality, there were no significant differences in the surgical duration, blood loss, length of hospital stay, or complications when compared to patients with unifocal PNET. However, the rate of total pancreatectomy was significantly higher in patients with multifocal tumors. Given that total pancreatectomy carries a higher risk of surgery and an increased rate of complications, and can lead to a poorer quality of life post-surgery compared to other procedures, various factors, including patient preferences, should be carefully considered when determining the treatment approach [12]. 

In this study, we discuss the optimal treatment for multiple tumors. Initially, since no significant difference in postoperative complications was observed between unifocal and multifocal tumors when employing the same surgical approach, the extent of surgery should be determined based on oncologic outcomes and post-surgery quality of life rather than the risk of complications. Oncologically, although multifocal tumors exhibited a higher recurrence rate compared to solitary tumors, there was no significant difference in long-term overall survival, and the prognosis remained generally favorable. This suggests that removing every lesion smaller than 2 cm in patients with multiple tumors does not significantly improve survival rates. Therefore, opting for the resection of lesions larger than 2 cm while cautiously monitoring those under 2 cm may better enhance post-surgical quality of life. However, given the higher recurrence rate in multifocal PNETs, it its crucial to anticipate a potentially higher recurrence rate, especially when smaller tumors are left unresected, necessitating more aggressive surveillance. Moreover, in cases where only NF-PNETs smaller than 2 cm are diagnosed as multiple, immediate surgical resection may not be essential. Nevertheless, given the higher recurrence rate compared to solitary NF-PNET, more aggressive surveillance is warranted.

This study had some limitations. Approximately 90% of patients were diagnosed with a single lesion, potentially impacting the statistical power due to the smaller number of patients with multifocal PNETs. The inclusion of patients with genetic disorders poses a challenge in adequately adjusting for the long-term prognostic impact of these conditions. Additionally, the study included tumors like NEMA, which are difficult to diagnose via conventional imaging preoperatively. Moreover, another limitation was the lack of adjustments between groups for additional interventions, such as liver-directed therapy, pancreas or liver resection, and chemoradiotherapy, in cases where recurrence was detected during follow-up after resection. Furthermore, before guidelines recommended observation for tumors smaller than 2 cm, patients with suspected PNETs underwent surgical resection as the primary treatment, regardless of the tumor size. Consequently, this study lacked uniform surgical indications across patients, which varied according to the timing of their surgery. Lastly, the current research, being a single-institution and retrospective study, had inherent limitations, indicating the need for future multi-institutional studies or prospective research with matched cohorts to validate and expand upon these findings.

## Conclusion

This study’s findings suggest that the presence of multiple tumors was associated with poorer recurrence-free survival but did not affect long-term survival following surgery. In the presence of multifocal tumors, targeting complete resection could necessitate a more extensive surgical approach. Despite the difference in multifocality, there were no significant differences in perioperative outcome and the risk of postoperative complications. Given the long-term oncologic outcome and quality of life following surgery, resection of tumors over 2 cm is advisable in patients with multifocal PNETs, while a cautious “wait-and-see” approach for smaller tumors (under 2 cm) can minimize the extent of resection and improve the quality of life. In cases with only small multifocal NF-PNETs (< 2 cm), immediate resection may not be crucial, but the higher recurrence rate than that in solitary NF-PNET necessitates intensified surveillance. The results underline the necessity for further research to optimize the management of multifocal NF-PNETs, reinforcing the need for tailored treatment approaches.

### Electronic supplementary material

Below is the link to the electronic supplementary material.

Supplementary Material 1

Supplementary Material 2

Supplementary Material 3

Supplementary Material 4

## Data Availability

Data is provided within supplementary information file.

## Abbreviations

ASA: American Society of Anesthesiologists
BMI: Body Mass Index
CI: Confidence Interval
CCI: Comprehensive Complication Index
CP: Central Pancreatectomy
DGE: Delayed Gastric Emptying
DP: Distal Pancreatectomy
ENETS: European Neuroendocrine Tumor Society
Hereditary SD: Hereditary Syndrome
HR: Hazard Ratio
NF-PNETS: Non-Functioning Pancreatic Neuroendocrine Tumors
MEN 1: Multiple Endocrine Neoplasia type 1
MIS: Minimally Invasive Surgery
NANETS: Neuroendocrine Tumor Society Consensus
NF-PNETS: Non-Functioning Pancreatic Neuroendocrine Tumors
OS: Overall Survival
PNETS: Pancreatic Neuro-Endocrine Tumors
POPF: Postoperative Pancreatic Fistula
PostOp LOS: Postoperative Length of Stay
PPPD: Pylorus-Preserving Pancreatico-Duodenectomy
RFS: Recurrence-Free Survival
SD: Standard Deviation
SMV: Superior Mesenteric Vein
VHL: Von Hippel-Lindau
WHO: World Health Organization
NEMA: Pancreatic Neuroendocrine Microadenomas
