# Supplementary figures and images for: Optimal surgical management of unifocal vs. multifocal NF-PNETs: a respective cohort study
Source: World J Surg Oncol. 2024 Apr 26;22:115. doi: 10.1186/s12957-024-03383-9 (PMC11046948; doi:10.1186/s12957-024-03383-9)

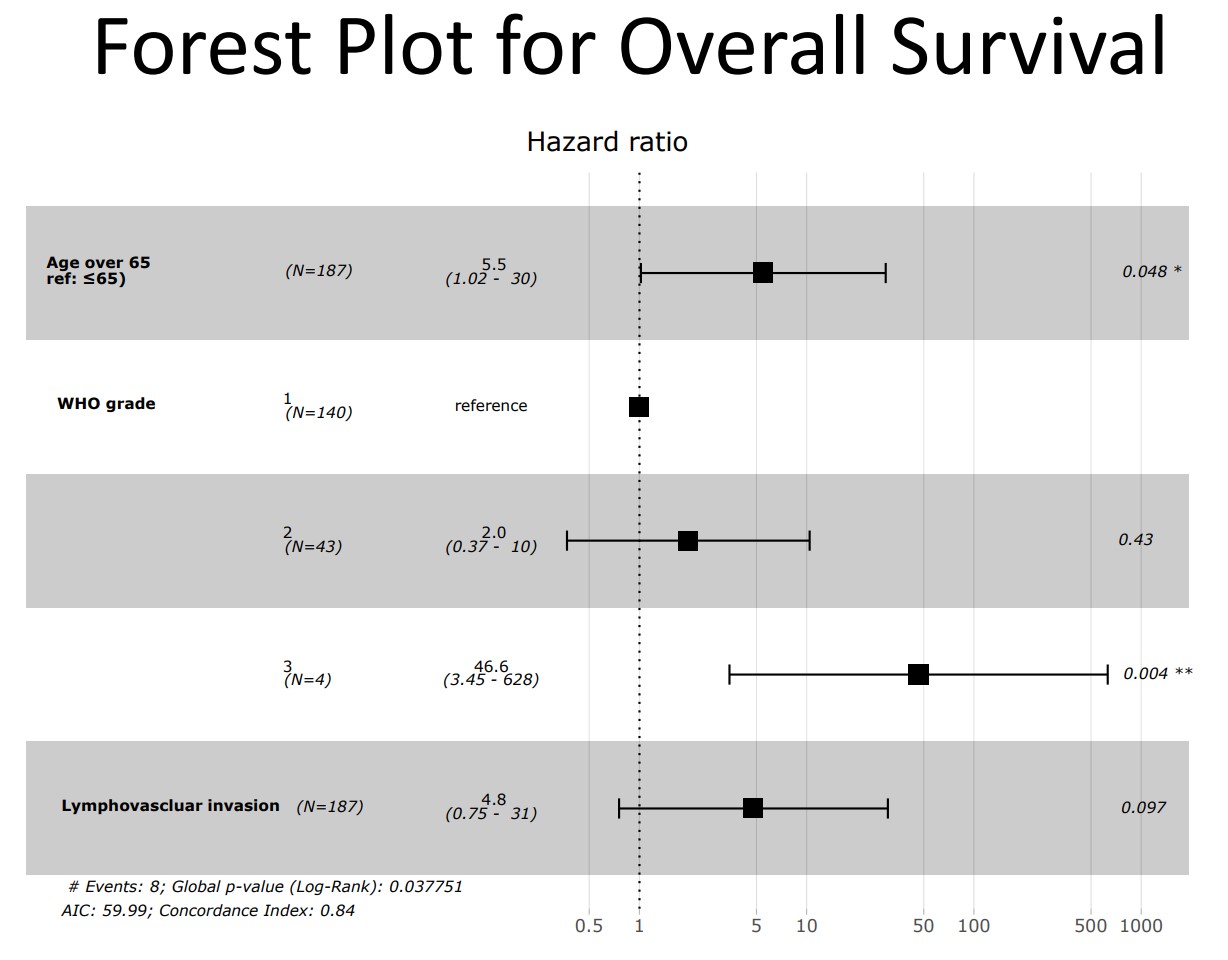

Supplement: Supplementary file 1 — Supplementary Material 1 [file 12957_2024_3383_MOESM1_ESM.jpg]

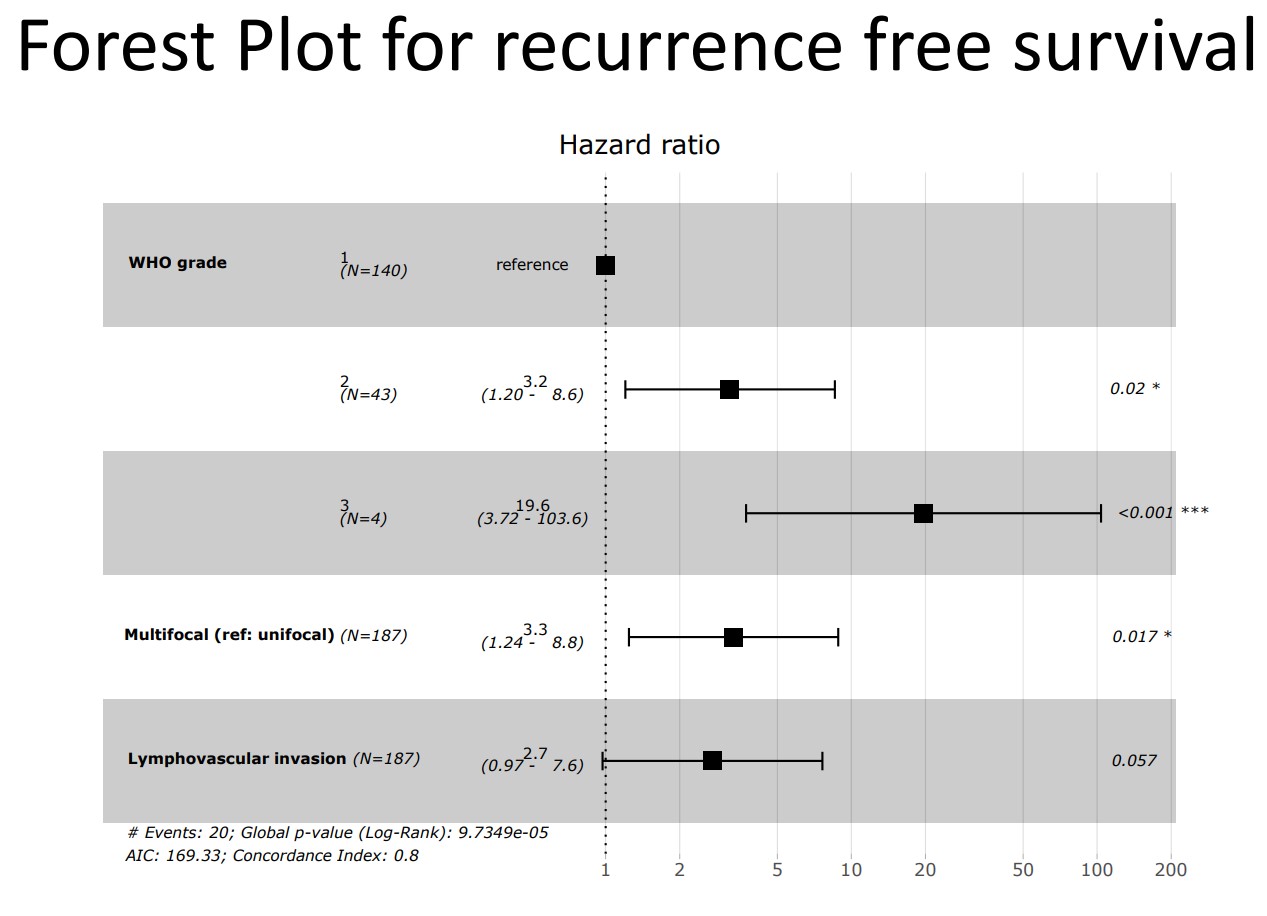

Supplement: Supplementary file 2 — Supplementary Material 2 [file 12957_2024_3383_MOESM2_ESM.jpg]
