# Supplementary material for: Optimal surgical management of unifocal vs. multifocal NF-PNETs: a respective cohort study
Source: World J Surg Oncol. 2024 Apr 26;22:115. doi: 10.1186/s12957-024-03383-9 (PMC11046948; doi:10.1186/s12957-024-03383-9)
Supplement: Supplementary file 4 — Supplementary Material 4 [file 12957_2024_3383_MOESM4_ESM.docx]

**Supplementary Table 1. Comparison of surgical outcomes between open pancreatectomy and MIS in patients diagnosed with NF-PNETs**

|  | open | MIS | p |
| --- | --- | --- | --- |
|  | (N=71) | (N=116) |  |
| Type of surgery |  |  | < 0.001 |
| Enucleation | 14 (19.7%) | 10 (8.6%) |  |
| Distal pancreatectomy | 21 (29.6%) | 72 (62.1%) |  |
| Central pancreatectomy | 4 (5.6%) | 5 (4.3%) |  |
| Pancreaticoduodenectomy | 26 (36.6%) | 22 (19.0%) |  |
| Total pancreatectomy | 6 (8.5%) | 7 (6.0%) |  |
| Operation time (min) | 258.0 [182.5;340.5] | 228.5 [157.0;374.0] | 0.243 |
| Estimated blood loss (cc) | 250.0 [100.0;600.0] | 100.0 [50.0;200.0] | < 0.001 |
| transfusion | 8 (11.3%) | 5 (4.3%) | 0.081 |
| Severe complication (over grade 3a) | 11 (15.5%) | 8 (6.9%) | 0.101 |
| Delayed Gastric Emptying |  | | 0.654 |
| Grade A | 8 (11.3%) | 9 (7.8%) |  |
| Grade B | 0 (0.0%) | 1 (0.9%) |  |
| Postoperative pancreatic fistula |  | | 0.826 |
| Biochemical leak | 23 (32.4%) | 34 (29.3%) |  |
| Grade B | 5 (7.0%) | 7 (6.0%) |  |
| Comprehensive complication index | 8.7 [0.0;20.9] | 0.0 [0.0;14.8] | 0.064 |
| Clavien-Dindo classification |  | | 0.077 |
| Grade 1 | 20 (28.2%) | 18 (15.5%) |  |
| Grade 2 | 9 (12.7%) | 20 (17.2%) |  |
| Grade 3a | 7 (9.9%) | 7 (6.0%) |  |
| Grade 3b | 2 (2.8%) | 1 (0.9%) |  |
| Grade 4 | 2 (2.8%) | 1 (0.9%) |  |
| Length of hospital stay (days) | 14.0 [11.0;25.5] | 9.0 [8.0;13.0] | < 0.001 |

**Supplementary Table 2. Patterns of recurrence following resection in patients with NF-PNETs**

|  | Unifocal | Multifocal | p |
| --- | --- | --- | --- |
|  | (N=169) | (N=18) |  |
| Recurrence pattern |  |  | 0.002 |
| Pancreas | 2 (1.2%) | 2 (11.1%) |  |
| Liver | 6 (3.6%) | 1 (5.6%) |  |
| Pancreas and liver | 3 (1.8%) | 0 (0.0%) |  |
| Regional lymph node | 2 (1.2%) | 0 (0.0%) |  |
| Distant metastasis | 1 (0.6%) | 3 (16.7%) |  |
